# Application of Reinforcement Learning Techniques in De Novo Drug Design: A Systematic Literature Review

**DOI:** 10.1002/hsr2.72132

**Published:** 2026-03-23

**Authors:** Masuda Begum Sampa, Nor Hidayati Abdul Aziz

**Affiliations:** ^1^ Faculty of Engineering and Technology Multimedia University Melaka Malaysia; ^2^ University of Science and Technology Chittagong (USTC) Chattogram Bangladesh; ^3^ Centre for Advanced Analytics, CoE for Artificial Intelligence Multimedia University Melaka Malaysia

**Keywords:** de novo drug design, drug discovery, reinforcement learning

## Abstract

**Background and Aims:**

De novo drug design is the process of generating novel lead compounds that possess desirable pharmacological activities and optimal physicochemical properties for therapeutic development. In recent years, it has evolved into a key computational strategy for discovering and optimizing new therapeutic compounds. Reinforcement learning (RL), a branch of artificial intelligence, has emerged as a powerful tool to address the complex, sequential decision‐making processes involved in molecular generation. This study aims to review recent applications of RL in de novo drug design, highlight commonly used algorithms, identify major challenges, and discuss future research directions.

**Methods:**

A systematic literature review (SLR) was conducted following standard review procedures. Articles published between January 2017 and January 2024 were retrieved from Google Scholar using the keyword “Reinforcement Learning Techniques in de novo Drug Design.” Studies were screened based on eligibility criteria, including relevance to RL‐based molecular generation, English language, and full‐text availability. Selected papers were analyzed to extract information on RL algorithms, design strategies, and application areas.

**Results:**

The reviewed studies demonstrate that RL has been successfully applied to molecular generation, optimization, and drug‐target design. Commonly used algorithms include policy‐gradient, actor–critic, and value‐based methods, often integrated with deep generative models such as recurrent neural networks (RNNs), variational autoencoders (VAEs), generative adversarial networks (GANs), and graph neural networks (GNNs). RL frameworks have optimized properties like binding affinity, solubility, and bioavailability, while promoting molecular diversity. Despite these advances, challenges remain in sample efficiency, reward formulation, and interpretability.

**Conclusion:**

Reinforcement learning provides a robust framework for automated drug design, enabling intelligent exploration of chemical space and the generation of novel, bioactive compounds. However, further improvements in multi‐objective optimization, computational efficiency, and model transparency are essential for broader clinical applicability. Future research should focus on hybrid RL architectures and explainable AI techniques to bridge computational and experimental drug discovery.

## 1 Introduction

Drug discovery in modern healthcare aims to create novel chemical compounds with therapeutic potential. However, identifying new molecules within the vast chemical space is both time‐consuming and expensive. Traditionally, researchers have relied on various computational and heuristic approaches such as molecular docking, quantitative structure–activity relationship (QSAR) modeling, and genetic algorithms to accelerate the search for promising compounds. These classical methods have provided valuable insights, but are often limited by their dependence on predefined chemical rules and limited exploration capabilities.

The need for more efficient and intelligent molecular design strategies has driven increasing interest in artificial intelligence (AI) for drug discovery. Among AI‐based approaches, deep generative models have shown particular promise in automating and optimizing molecular generation [1], thereby reducing the risk of failure during preclinical and clinical stages [2].

Within this context, de novo drug design refers to the use of computational techniques to automatically generate new chemical compounds with predefined desirable molecular properties [2]. The objective is to construct compounds that fit specified structural or physicochemical requirements, such as binding affinity, solubility, or bioavailability. De novo design provides a systematic framework for exploring chemical space and identifying molecules that may not exist in current databases.

RL has become one of the most widely used techniques for molecule generation (Hu et al., 2024). Among various AI methods, RL has emerged as a powerful paradigm for de novo drug design due to its capacity to handle sequential decision‐making problems. In RL, an agent interacts with an environment, takes actions, and learns optimal strategies through rewards and penalties associated with outcomes [3]. The process is typically modeled as a Markov Decision Process (MDP) with defined states, actions, and rewards [2].

In molecular generation, the RL agent explores the chemical space, making sequential decisions to construct valid molecular structures. Deep neural networks, often implemented within RNN or transformer architectures, serve as the agent responsible for generating molecules as one‐dimensional simplified molecular input line entry system (SMILES) strings. Reward functions guide the agent toward molecules with optimal physicochemical or pharmacological properties [4].

RL techniques can be broadly divided into value‐based and policy‐based methods. Value‐based approaches aim to estimate a value function that represents the expected return from a given state, while policy‐based methods directly learn the optimal policy [5]. Recent studies have also introduced auxiliary loss functions to promote molecular diversity and exploration [6]. By framing molecule generation as a sequential decision‐making process, RL enables iterative optimization of compounds toward multiple desired targets, demonstrating its growing potential in drug design and discovery [7].

This review provides a comprehensive analysis of how reinforcement learning has evolved over the last 5 years in de novo drug design. While previous reviews have addressed AI's broader role in drug development, they often lack detailed insights into generative and reinforcement learning‐based approaches. To address this gap, this review focuses on three key research questions:

1. Which reinforcement learning algorithms are used to generate new molecular structures?
2. What types of tasks have been addressed using RL techniques?
3. What are the future research directions for RL in de novo drug design?

### 1.1 Basics of Reinforcement Learning

RL encompasses a set of machine learning techniques focused on training an agent to make decisions within a specific environment by learning optimal actions through trial and error. The agent receives positive rewards for actions that align with the desired objective and negative feedback for those that do not. In the context of molecular generation, the agent functions as the molecular generator. It sequentially constructs molecular structures by selecting chemical building blocks or molecular tokens with the objective of maximizing the reward signal. In generative molecular design, RL is used to guide a model toward producing compounds that meet certain property criteria. This approach has become a popular optimization strategy in drug discovery due to its ability to iteratively improve molecule generation based on predefined goals [8]. The four basic components of RL are as follows:

1. State representation
  In the agent‐environment RL interface, the state can be any information available to the agent. The state should be defined by the Markov property. That means, the state signal is not supposed to convey all the information about the environment to the agent, but it should summarize past information such that all relevant information is not missed. A state signal with this property is called Markov [9]. In the context of molecular generation, the state represents the current partial or complete molecular structure during the generation process. It encodes information about the molecular configuration, which the agent uses to determine the next modification or addition to the molecule.
2. Policy Optimization.
  When states are formulated, it is the policy that determines which action to take in each state. For policy optimization, various RL algorithms have been utilized. Before the advent of deep reinforcement learning (DRL), RL methods could be generally classified into tabular and approximate methods. Tabular methods include policy iteration, Q‐learning, Sarsa, Sarsa (*λ*), R‐learning, and MCTS. Approximate methods include fitted Q and gradient value iteration. On the other hand, DRL methods could be generally divided into three groups: value‐based (DQN), policy gradient (REINFORCE and REINFORCE‐wb), and actor‐critic (DDPG and PPO) methods.
3. Reward Formulation.
  The reward signal from the environment reflects how good or bad the agent is performing through selecting actions. Therefore, designing an informative reward signal is critical for success/learning of the agent. In fact, in RL, the reward signal is the only way to tell the agent what to do, not how to do it. In general, defining a proper reward function is a hard problem, and it is more of a trial‐and‐error or engineering process. There is no definite rule to design a good reward function in a specific problem.
4. Environment Building.
  The environment represents the molecular design space and evaluation system. It receives the molecular structures generated by the agent and evaluates them based on predefined criteria such as chemical validity, drug‐likeness, binding affinity, or synthetic accessibility. Building a suitable environment to properly train and evaluate the agent in medical imaging is challenging.

To illustrate the concept of a Markov decision process (MDP) in a simple and intuitive manner, consider the task of finding the root of a basic mathematical function, such as f(x) = x − 3. In this case, the objective is to identify the value of x for which the function evaluates to zero, i.e., f(x) = 0. This scenario can be modeled as an MDP with the following components: the state represents the current guess for the value of x; the actions available to the agent include increasing x by 1, decreasing x by 1, or keeping x unchanged; and the reward structure is defined such that the agent receives a positive reward (e.g., +10) if the correct root is found (f(x) = 0), and a small negative reward (e.g., −1) otherwise. For example, if the agent starts with a guess of x = 1, applies the “increase by 1” action twice, it reaches x = 3, where f(3) = 0, thus receiving the maximum reward. Over time, by exploring different guesses and observing the rewards, the agent learns the optimal sequence of actions to efficiently find the root.

**Figure 1:**
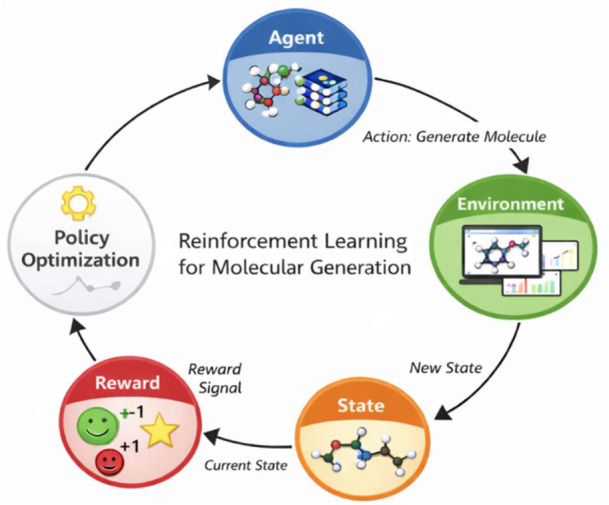
Reinforcement learning for molecular generation.

In the context of de novo drug design, RL is used to generate novel molecular structures with desirable pharmacological properties. The interaction among the key RL components guides the iterative optimization of molecular candidates. The following Figure 1 presents the interdependence among the key components of RL.

## 2 Material and Methods

### 2.1 Systematic Literature Review

**Figure 2:**
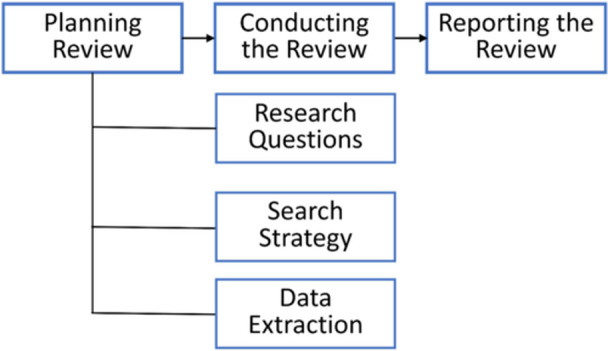
Steps of the review.

This study follows standard SLR procedures, including three main stages: (1) Planning the review, (2) Conducting the review, and (3) Reporting the review (Figure 2).

### 2.2 Articles Searching

We chose articles published on Google Scholar from January 2017 and January 2024. Most papers on the reinforcement learning application were published from 2017 onward. The papers went through a rigorous review procedure. “Reinforcement Learning Techniques in de novo Drug Design” was our search term. Not all papers are accessible to us. Thus, we looked over the articles that we were able to access on purpose.

### 2.3 Eligibility Criteria

Because machine learning methods contrived to generate novel molecules automatically have only recently expanded, 5 years was deemed appropriate for the present review. For example, the de novo drug design software design of genuine structures (DOGS), introduced in 2012, does not utilize any generative machine learning technique, although it would ultimately inspire subsequent experiments. Until 2017, the explicit application of generative molecular models to de novo pharmacology was not stated. Considering the relative novelty of this field, the selected period was chosen to accommodate the sparsity of relevant content before 2017. Only articles found to satisfy the following criteria were included in the review:

1. The study is written in English.
2. The study was published between January 2017 and January 2024.
3. The study is a full‐text research article or review.
4. The study concerns the use of reinforcement learning to generate small, drug‐like molecules de novo.

Articles that did not explicitly address our 3 research questions were excluded from the review. Review articles were also excluded.

### 2.4 Software and Tools Used in the Reviewed Studies

Python is widely used as the programming language for implementing RL algorithms due to its simplicity, flexibility, and extensive ecosystem of scientific libraries. It offers powerful machine learning frameworks such as TensorFlow and PyTorch, which facilitate the design and training of complex neural network architectures commonly employed in RL agents. Additionally, Python provides specialized libraries like OpenAI Gym for standardizing RL environments and Stable Baselines3 for access to state‐of‐the‐art RL algorithms, enabling rapid prototyping and experimentation. In the context of molecular generation and optimization, Python's integration with cheminformatics tools and data processing libraries streamlines the workflow, from environment setup and agent training to evaluation and visualization. This makes Python the preferred choice for researchers developing RL‐based drug discovery and molecular design systems.

## 3 Findings

Which reinforcement learning algorithms are used to generate new molecular structures?

RL has emerged as a powerful strategy for optimizing molecular properties and generating novel compounds [10]. In molecular design, RL treats molecule generation as a sequential decision‐making process, where an agent constructs new molecules step by step, optimizing characteristics such as binding affinity, solubility, and toxicity. The SMILES sequence building process can thus be viewed as a series of decision‐making steps, where the model determines the optimal token at each iteration [11].

A wide variety of RL algorithms have been applied to molecular generation. Actor–critic frameworks are frequently used due to their stability and balance between exploration and exploitation. Tang et al. [1] introduced EarlGAN, an actor–critic RL agent‐driven GAN that integrates long short‐term memory (LSTM) based bidirectional generators and discriminators. EarlGAN successfully generates chemically valid and diverse molecules, maintaining a strong balance between novelty, validity, and chemical property distribution. Similarly, [2] adopted an actor–critic model to produce novel compounds, while [12] further demonstrated its ability to simultaneously optimize multiple drug design parameters using fragment‐based reinforcement learning.

Policy‐gradient‐based algorithms have also proven effective. Popova et al. [3] developed reinforcement learning for structural evolution (ReLeaSE), which combines generative and predictive models trained jointly with RL to bias molecular generation toward desired bioactive and physicochemical properties. Pereira et al. [7] applied a policy‐gradient algorithm to optimize drug candidates selective for the adenosine A2A receptor while ensuring permeability and solubility across the blood‐brain barrier. The policy gradient for forward synthesis (PGFS) approach proposed by [13] embeds synthetic accessibility into the design process by training the RL agent to apply valid chemical reactions to initial commercial molecules, creating a synthetically feasible exploration of chemical space.

Advanced RL architectures have also integrated deep generative models. Wang et al. [4] proposed an A2C‐based framework for molecular design, while [14] combined a graph attention model with RL to learn protein active sites and generate target‐specific molecules. Hu et al. [6] applied multiple GPT agents for molecular generation using RL optimization, illustrating the trend toward transformer‐based architectures. Korshunova et al. [10] combined policy‐gradient, fine‐tuning, and experience replay techniques, treating the generative model as a policy network that predicts the next SMILES token, effectively adapting RL for large‐scale molecular synthesis. Du et al. [15] developed a data‐free, structure‐based RL framework that integrates Monte Carlo tree search (MCTS) with policy and value networks to generate molecular structures directly within protein binding pockets. In this framework, MCTS serves as the core decision‐making mechanism, efficiently exploring the three‐dimensional chemical space, while the policy network guides molecular modification actions and the value network estimates the quality of partial structures. This combination allows the system to balance exploration and exploitation without relying on pre‐existing molecular datasets, enabling de novo molecule generation guided by structural and energetic feedback. Such integration of MCTS with deep RL represents an important step toward data‐independent and structure‐aware drug design frameworks.

Other approaches combine RL with graph‐based generative models. Atance et al. [16] proposed an RL framework to fine‐tune graph GNNs, where the agent learns to modify molecular graphs to optimize a reward function reflecting target properties such as drug‐likeness or bioactivity. McNaughton et al. [17] implemented an actor–critic reinforcement learning framework to drive de novo molecule generation, with the actor generating 3D molecular scaffolds incrementally and the critic—a GNN—evaluating partial molecules for binding probability to a target protein. Liu et al. [18] explicitly applied a policy‐gradient RL algorithm combined with Pareto‐based multi‐objective optimization, enhancing diversity and exploration via mutation and crossover networks alongside the generative agent. Similarly, [19] used a policy‐gradient method in REINVENT 2.0, where an LSTM‐based generative model was trained to maximize a reward function representing desired molecular properties, iteratively improving molecule generation by learning which token sequences lead to higher rewards.

What types of tasks have been addressed using RL techniques?

Beyond de novo molecular generation, RL has been employed across a diverse range of molecular optimization tasks, including improving the pharmacological and structural properties of existing compounds through iterative chemical modifications and feedback assessment. In disease‐specific drug discovery [13], used two pre‐trained VAEs one encoding transcriptomic profiles and the other generating molecular structures—to design anticancer drugs optimized via RL. The reward function was based on predicted drug sensitivity (IC50), enabling the creation of compounds with targeted efficacy against specific cancer cell types. Extending this concept [20], integrated gene expression profile (GEP) data with RL and deep generative models, facilitating the generation of compounds effective in particular disease contexts.

RL has also been coupled with simulation‐based molecular evaluation. Wang et al. [21] combined docking simulations with deep RL, wherein agents iteratively modified molecular structures based on predicted biological activity and binding affinity. Similarly [16], leveraged graph‐based deep generative models fine‐tuned via RL to design compounds with enhanced drug‐likeness and predicted activity for DRD2 receptors, achieving up to 95% predicted bioactivity. This RL framework was applied to multiple structure‐based drug design tasks, successfully generating novel 3D molecular structures within target protein binding pockets and optimizing them for binding affinity, drug‐likeness, and scaffold diversity. Du et al. [15] further validated their approach across different protein targets, demonstrating its robustness and generalization capability. These studies illustrate that RL can efficiently address multiple objectives in de novo drug design, including molecular generation, affinity optimization, and chemical diversity enhancement.

Reinforcement learning has also been used to control molecular size, optimize drug‐likeness (QED), and enhance bioactivity against specific targets such as the DRD2 receptor. These frameworks have effectively guided generative models to meet these objectives, achieving up to 95% predicted active compounds for DRD2 and surpassing previous benchmarks [16]. McNaughton et al. [17] focused on designing target‐specific inhibitors by growing molecules in three dimensions from a starting scaffold to fit specific protein binding pockets. Their RL setup optimizes multiple objectives simultaneously, including binding affinity (or binding probability) to the target, predicted potency, and synthetic accessibility. As a proof‐of‐concept, they applied their method to the main protease of SARS‐CoV‐2, demonstrating that RL‐based generative frameworks can produce molecules tailored to therapeutically relevant targets.

Liu et al. [18] addressed several key molecular design tasks using RL, most prominently polypharmacology, aiming to generate molecules with high affinity for multiple therapeutic targets simultaneously. In DrugEx v2, the model was trained to optimize interactions with the A₁ and A₂A adenosine receptors while reducing predicted off‐target activity toward the hERG potassium channel. Beyond multi‐target design, the framework was applied to broader goal‐directed molecular optimization tasks, including improving physicochemical properties, enhancing drug‐likeness, increasing molecular diversity, and tackling complex challenges from the GuacaMol benchmark, such as scaffold matching and similarity‐based optimization.

In REINVENT 2.0, the RL framework has been applied to address exploration and exploitation challenges in chemical space [19]. The system allows users to define a multi‐parameter scoring function that guides molecule generation according to desired properties. It explicitly supports exploration mode, favoring structurally novel compounds, and exploitation mode, generating molecules similar to a specified region of interest. Additionally, a diversity filter is applied during training to penalize redundancy and encourage chemical diversity.

What are the future research directions for RL in de novo drug design (as suggested in the reviewed articles)?

Although RL‐based molecular design has demonstrated significant potential, several avenues remain for further advancement. Future research should focus on improving reward formulations that integrate multi‐objective optimization criteria, balancing novelty, drug‐likeness, efficacy, and synthetic feasibility. Incorporating environmental feedback from biological assays and simulations could further enhance the biological relevance of generated compounds.

A key direction involves unifying generative and predictive modeling within a single RL framework. While studies such as [3] and [20] have begun exploring this integration, broader adoption of multi‐agent and hierarchical RL frameworks may enhance the agent's ability to explore complex chemical spaces efficiently. The use of transformer‐based agents [6] also represents a promising path toward more scalable and interpretable models. Additionally, frameworks employing transfer learning and experience replay [10] could enable knowledge reuse across drug classes, reducing data requirements and improving generalization. Establishing structured benchmarks for evaluating RL methods in de novo drug design would also facilitate more consistent comparisons and accelerate progress in the field.

Improving the sample efficiency of RL models is another critical challenge. Many RL frameworks rely on reward signals derived from computationally intensive evaluations, such as molecular docking, molecular dynamics, or quantum chemical simulations. While accurate, these evaluations are computationally costly, limiting the scalability of RL models. Enhancing sample efficiency through model‐based RL, surrogate modeling, or transfer learning could substantially reduce computational demands while maintaining performance. Moreover, the exploration–exploitation trade‐off remains a key issue: excessive exploration may generate chemically invalid or redundant candidates, whereas overly exploitative policies risk premature convergence to suboptimal solutions. Adaptive exploration strategies, such as uncertainty‐guided or curiosity‐driven mechanisms, may help balance these competing objectives and enable more robust molecular discovery pipelines. Du et al. [15] highlighted directions for future work, including improving the accuracy of molecular scoring, extending frameworks to broader sets of protein targets, enhancing computational efficiency, and integrating 3D‐MCTS RL frameworks with other drug design strategies. Building on these insights, future studies could incorporate multi‐objective optimization to balance binding affinity, pharmacokinetics, and toxicity, include experimental feedback to improve translational reliability, and combine RL frameworks with modern generative models to enhance molecular diversity and exploration efficiency. Together with high‐performance computing, these approaches could make RL‐driven de novo drug design more scalable, robust, and experimentally relevant.

Addressing the current limitations of existing models is also critical. The high computational cost and time required for pre‐training GraphINVENT‐like models, along with the tendency to generate undesirable macrocycles or unstable moieties when designing larger molecules, remain key challenges. Optimizing non‐linear properties, such as QED, is difficult due to the limited examples of large molecular subgraphs during pre‐training. Future work could focus on improving scoring functions to better estimate molecular stability, drug‐likeness, and synthetic accessibility, thereby reducing the generation of unstable or impractical molecules. Exploring more efficient pre‐training strategies, leveraging transfer learning for multiple tasks, and integrating RL with other generative approaches could further enhance the design of high‐quality, drug‐like molecules [16].

McNaughton et al. [17] highlight additional promising directions, including refining multi‐objective reward functions to balance binding potency, synthetic feasibility, solubility, and other drug‐like properties. They also advocate applying the 3D‐Scaffold RL framework to a broader set of protein targets to assess generalizability and robustness, increasing interpretability by analyzing how specific atomic placements influence binding or drug‐like metrics, and bridging computational design with wet‐lab experiments to validate predictions of binding affinity, bioactivity, and synthetic accessibility. Liu et al. [18] emphasize incorporating additional molecular objectives, such as synthetic accessibility and molecular stability, into multi‐objective reward functions, enabling flexible user control over conflicting optimization goals, and extending DrugEx with advanced neural architectures, including transformers, graph‐based generative models, and fragment‐based approaches. Expanding and refining Pareto‐based optimization strategies will also be important for maintaining chemical diversity while meeting increasingly complex pharmacological criteria. Blaschke et al. [19] suggest integrating more advanced predictive models into reward functions, accommodating richer molecular representations, and improving interpretability and controllability of the generative process. They also highlight combining REINVENT with complementary molecular design strategies and validation pipelines to increase the applicability of RL‐generated molecules in real‐world drug discovery.

## 4 RL Drug Design Framework

We identified four standard components after carefully studying all collected reinforcement learning drug discovery studies. A compelling reinforcement learning drug discovery study should meticulously design and address these components. To standardize the development process of RL drug discovery, we propose a framework comprising four key elements: (1) State Representation, (2) Policy Optimization, (3) Reward Formulation, and (4) Environment Building.

State Representation: State representation determines how molecular information is captured and interpreted by the agent, directly influencing its ability to explore chemical space effectively. The set of states S is defined as all possible strings in the alphabet with lengths from zero to some value T. The initial state is defined as the unique state s_0_ of length 0. Because it ends training, the state s_T_ of length T is called the terminal state. The terminal states set is the subset of terminal states S* = {s_T_ ∈ S}of S, including all states s_T_ of length T [3]. The initial state for the policy gradient in reinforcement learning was the RNN model trained on the ZINC data set. 10,000 SMILES sequences were generated for performance evaluation once the RL and model training processes had converged [11]. Therefore, State representations should integrate molecular graphs and physicochemical descriptors for richer feature encoding.

Policy optimization: Policy optimization governs the agent's learning strategy, ensuring efficient exploration and exploitation in molecule generation. In reinforcement learning, every RL algorithm works as the agent. The policy is the tool that maps the present state to the probability distribution for selecting the following action [7]. Policy or strategy is used to generate new molecules. The policy approximation model estimates the probability distribution p(at | st‐1) of the subsequent action using the prior state, st‐1, as an input. A sample of the following action is taken from this predicted likelihood. The study [7], the REINFORCE algorithm is used for policy optimization. If the states and actions used to train the agent are generated by the agent, the learning is referred to as on‐policy. Conversely, the learning is called off‐policy if they are generated by other means [5]. Therefore, Policy optimization can benefit from hybrid on‐policy/off‐policy algorithms (e.g., PPO or DDPG) to balance stability and adaptability.

Reward Formulation: Reward formulation translates drug‐likeness and pharmacological objectives into quantifiable feedback signals, guiding the agent toward chemically meaningful outcomes. Reward r(s_T_) is a function of the predicted property of s_T_ using the predictive model P [3]. Therefore, Reward functions should incorporate multiple objectives such as binding affinity, solubility, and synthetic feasibility.

Environment Building: Environment Building provides the simulated experimental setup that reflects real‐world molecular interactions and determines the reliability of the agent's training process. RL describes and solves problems by having agents interact with their environment and use learning strategies to maximize returns or achieve specific goals [22]. The proposed environment for drug discovery offers a highly challenging test‐bed for RL algorithms due to its large state space and high‐dimensional continuous action space with hierarchical actions [23]. Therefore, Environment building should aim to simulate realistic chemical environments, incorporating docking or molecular dynamics feedback for more accurate learning.

Actions: The agent selects from a set of actions [7]. Since the whole set of letters and symbols is used to build the canonical SMILES strings, which are most frequently employed to encode chemical structures—the set of actions A is defined as an alphabet. As an illustration, the molecule of aspirin is encoded as [CC(O)OC1CCCCC1C(O)O] [3]. The policy π, where π(a, s) = P(a_t_ = a | s_t_ = s), is used to determine the action that the agent chooses at time t [2].

## 5 Discussion

Reinforcement learning remains one of the most widely used techniques for molecular generation, alongside genetic algorithms, diffusion‐based models, and flow network–based generative approaches. Evidence suggests that traditional strategies such as genetic algorithms can perform as well as or even better than modern machine learning models for specific optimization tasks, such as log P optimization [24]. Recent advances in generative modeling have further expanded the capabilities of de novo drug design. For instance, [25] proposed Diffusion Structure‐Based Drug Design (DiffSBDD), an SE(3) equivariant 3D diffusion model capable of generating novel ligands conditioned on the three‐dimensional structure of protein pockets. This method enables the design of diverse, drug‐like molecules with high docking scores and supports flexible optimization operations such as scaffold modification and fragment growing, underscoring the potential of diffusion models to complement RL in structure‐based drug design.

In parallel, new generative paradigms have emerged as alternatives or complements to reinforcement learning. Flow Network–based Generative Models (GFlowNets) [26] learn to sample molecular structures with probabilities proportional to their reward values, facilitating the generation of diverse high‐quality candidates rather than collapsing toward a single optimal solution. Unlike conventional RL, which often gravitates toward local reward maxima, GFlowNets promote diversity‐oriented exploration of chemical space, a key advantage in drug discovery where multiple structurally distinct molecules with comparable activity may be desired.

Gao et al. [27] further emphasized the limitations of existing RL‐based molecular design methods through the practical molecular optimization (PMO) benchmark. Their findings show that many RL models exhibit poor sample efficiency under realistic oracle constraints, with established approaches such as REINVENT outperforming several newer methods when evaluation budgets are limited. These insights highlight the necessity of assessing RL algorithms not only on final optimization outcomes but also in terms of computational efficiency—an essential consideration for practical, resource‐constrained de novo drug design workflows.

### 5.1 Challenges, Dilemmas, and Unresolved Concerns

Despite the considerable promise of RL in de novo drug design, several persistent challenges and unresolved concerns continue to limit its routine adoption in practical drug discovery pipelines. One major limitation is sample inefficiency, as RL models often require the evaluation of large numbers of candidate molecules to converge toward optimal solutions. This issue becomes particularly pronounced when reward functions rely on computationally expensive procedures such as molecular docking, molecular dynamics simulations, or free‐energy calculations, substantially increasing computational cost and limiting scalability [5].

Another fundamental challenge involves the exploration–exploitation trade‐off. Excessive exploration may result in the generation of invalid, redundant, or chemically implausible molecular structures, whereas excessive exploitation can lead to premature convergence toward locally optimal compounds with limited structural diversity. Managing this balance remains nontrivial, especially in high‐dimensional chemical spaces where meaningful exploration is inherently difficult [3].

The design of effective reward functions represents a further unresolved dilemma. In practice, reward functions must simultaneously account for multiple, often competing objectives, including drug‐likeness, predicted binding affinity, toxicity, pharmacokinetic properties, and synthetic feasibility. However, many existing RL implementations prioritize readily computable objectives—such as predicted affinity or QSAR scores—while inadequately capturing experimental constraints [28]. As a result, optimized molecules may perform well in silico but lack practical viability.

A closely related challenge in RL‐driven de novo drug design concerns the formulation of reward functions and their limited alignment with experimental feasibility. Many RL‐based molecular optimization frameworks rely on readily computable surrogate objectives—such as predicted binding affinity, QSAR scores, or physicochemical property metrics, which, while computationally efficient, may not adequately capture real‐world synthetic and stability constraints. For example, the benchmarking study by [29] highlights that generative models are frequently optimized against easily measurable properties, potentially overlooking practical medicinal chemistry considerations. Empirical evaluation of synthesizability further underscores this gap. Gao, Coley [30] demonstrate that molecules generated by state‐of‐the‐art generative models can achieve high benchmark scores yet remain challenging or impractical to synthesize when assessed using computer‐aided retrosynthetic planning tools. These findings indicate that RL‐generated compounds may exhibit structurally complex or synthetically inaccessible motifs that complicate laboratory validation and scale‐up. The limited integration of retrosynthetic analysis, stability‐aware constraints, and manufacturability considerations within current RL frameworks, therefore, widens the gap between computational optimization and experimental feasibility.

The interpretability of RL‐based molecular design models remains an unresolved concern, as many deep learning systems operate as black boxes that provide limited mechanistic insight into why specific molecular modifications are preferred, thereby restricting transparency and trust in industrial drug discovery environments [31, 32].

RL‐based molecular design frameworks are typically optimized using task‐specific reward functions that bias generative models toward desired properties within a limited chemical domain [5]. As a result, trained models may exhibit reduced transferability when applied to new biological targets or structurally distinct compound libraries due to domain‐specific optimization and variability in training data distributions [33]. This limited generalizability often necessitates retraining for different drug discovery tasks, thereby reducing policy reusability and increasing development cost.

Importantly, although numerous studies report strong computational performance using RL‐based molecular generation approaches [3, 5, 34], relatively few contributions include experimentally validated bioactive compounds. Most published methods remain at the proof‐of‐concept stage, focusing primarily on optimizing in silico objectives such as predicted activity, similarity metrics, or physicochemical properties rather than demonstrating downstream synthesis and biological confirmation. Benchmarking and review studies have explicitly highlighted this limitation, noting that high performance on computational tasks does not necessarily translate into practical impact in medicinal chemistry workflows [29, 35]. This persistent gap between algorithmic innovation and experimental validation underscores a critical challenge in the field, emphasizing the need for closer integration between generative modeling frameworks and real‐world drug discovery pipelines.

Given these limitations, it is essential to maintain realistic expectations regarding the current capabilities of RL‐driven molecular generation, particularly for a broad and interdisciplinary audience. At present, these methods are best viewed as decision support and hypothesis‐generation tools that can guide molecular exploration and prioritize promising candidates, rather than as autonomous systems capable of replacing medicinal chemistry expertise or experimental screening pipelines. Bridging the gap between computational frameworks and experimentally validated drug candidates will require advances in reward design, improved integration of synthetic planning and stability constraints, enhanced model interpretability, and closer collaboration between computational and experimental disciplines.

### 5.2 Authors' Proposed Future Research Directions

The following list of prospective research directions is based on the reviewed papers:

i. Future research may utilize big data as a lead set for training RL agents in molecular design. This approach enables RL agents to learn general rules and transformations from diverse molecular structures, enhancing the properties of molecules.
ii. In the field of applied drug development, carrying out focused, in‐depth studies on specific goals can help us make better use of task‐specific information, which will ultimately result in the design of candidate drug molecules that are more successfully developed.
iii. There is potential for improvement by integrating faster, more accurate molecular property prediction software and modifying term coefficients in the scoring function's design, especially in multi‐property applications [6].
iv. Implementing more reliable prediction models to filter newly generated compounds will be the main focus of future studies. To evaluate the effectiveness of potential medications, we intend to investigate the 3D data of the compounds and the related drug‐target complexes in the future [7].

### 5.3 Limitations of the Current Review Study

Despite its comprehensive scope, this review has several limitations. First, the coverage is limited to studies published on Google Scholar from January 2017 and January 2024, so recent developments in reinforcement learning for de novo drug design may not be captured. Second, the search was confined to selected databases and English‐language publications, introducing potential database and language biases that may have excluded relevant research. Third, the included studies exhibited substantial heterogeneity in tasks, datasets, and evaluation metrics, which precluded quantitative synthesis (e.g., meta‐analysis) and limited the review to qualitative assessment. At the study level, important limitations in the current body of research were identified. Many studies relied on small or single‐center datasets, restricting generalizability. Reproducibility was limited, as few studies made code or data publicly available. Computational considerations, including training time and hardware requirements, were rarely reported, despite being crucial for real‐world feasibility. While some studies addressed clinical relevance, large‐scale validation and integration into routine clinical practice remain limited. Methodologically, the review was restricted to publications from January 2017 and January 2024, and the formal search was conducted only in Google Scholar, some relevant studies may have been missed. Finally, while the review proposes an RL drug design framework and provides conceptual comparisons with existing studies, these remain interpretive rather than empirically validated, and future work is needed to test the framework in practical settings.

## 6 Conclusion

RL has emerged as one of the most promising methodologies for de novo drug design, offering a dynamic framework to accelerate and enhance the molecular discovery process. Through its ability to iteratively optimize chemical structures based on learned reward functions, RL enables the design of novel molecules with improved pharmacological properties such as binding affinity, solubility, and drug‐likeness. The reviewed studies demonstrate that RL algorithms—particularly policy‐gradient, actor–critic, and value‐based methods—have been successfully integrated with deep generative architectures, including RNNs, VAEs, GANs, and graph neural networks to explore chemical space more efficiently and with greater structural diversity. Despite these advances, several limitations remain. Current RL models often face challenges in balancing exploration and exploitation, handling sparse or delayed rewards, and achieving high sample efficiency when rewards are computationally expensive to calculate. Moreover, scalability and interpretability continue to pose obstacles to real‐world applications. Addressing these issues will require integrating RL with more data‐efficient learning strategies, hybrid optimization frameworks, and domain‐specific knowledge of molecular chemistry. Therefore, RL has demonstrated substantial potential for guiding molecular design and optimization, yet its effectiveness depends on improving sample efficiency, refining reward formulations, and enhancing model interpretability. Future research should focus on developing more robust, scalable, and explainable RL frameworks that can bridge the gap between in silico molecular generation and experimental drug discovery.

## Author Contributions

**Masuda Begum Sampa:** conceptualization, methodology, data curation, writing — original draft, writing — review and editing. **Nor Hidayati Abdul Aziz:** supervision, funding acquisition.

## Conflicts of Interest

The authors declare no conflicts of interest.

## Transparency Statement

The lead author Masuda Begum Sampa affirms that this manuscript is an honest, accurate, and transparent account of the study being reported; that no important aspects of the study have been omitted; and that any discrepancies from the study as planned (and, if relevant, registered) have been explained.

## Data Availability

The authors confirm that all data supporting the findings of this study are available within the article and its references. No additional datasets were generated or analyzed during the current study.
